# The Associations of Parenting Factors with Adolescent Body Mass Index in an Underserved Population

**DOI:** 10.1155/2013/715618

**Published:** 2013-05-15

**Authors:** Elizabeth M. Schneider, Dawn K. Wilson, Heather Kitzman-Ulrich, Sara M. St. George, Kassandra A. Alia

**Affiliations:** ^1^Department of Clinical & Health Psychology, University of Florida, P.O. Box 100165, Gainesville, FL 32610, USA; ^2^Department of Psychology, University of South Carolina, Columbia, SC 29208, USA; ^3^Texas Prevention Institute, University of North Texas Health Science Center, Fort Worth, TX 76107, USA

## Abstract

*Background*. The current study examined parental factors related to risk of adolescent obesity within the context of a family systems framework. *Methods*. Seventy predominantly African American, low-income caregiver-adolescent dyads participated in the study. Validated measures of parental perceived child risk for development of type 2 diabetes mellitus, parental limit setting for sedentary behavior, and parental nurturance were evaluated as predictors of adolescent body mass index. *Results*. In this cross-sectional study, multiple linear regression demonstrated that parents of adolescents with higher zBMI reported worrying more about their child's risk of developing type 2 diabetes mellitus. Parent limit setting was also a significant predictor of adolescent zBMI. Contrary to expectations, higher levels of nurturance were associated with higher adolescent zBMI. Post hoc analyses revealed a trend towards a significant interaction between nurturance and limit setting, such that high levels of both parental nurturance and limit setting were associated with lower adolescent zBMI. *Conclusions*. Current findings suggest the importance of authoritative parenting and monitoring of adolescent health behaviors in the treatment of obesity.

## 1. Introduction

Obesity has long been a major health concern among adults but more recently has become a public health priority among children and adolescents due to the increasing prevalence rates and associated health risks over the last three decades. Over 32% of children and adolescents in the United States are classified as overweight or obese, with the highest rates among ethnic minorities [1, 2]. Obesity places children at greater risk for a number of physical and mental health conditions including type II diabetes mellitus (T2DM) [3]. However, the factors that determine childhood body weight are still not completely understood. Though it is clear that energy intake and energy expenditure are under genetic influence, it is also clear that genetic factors do not fully explain the current increases in the prevalence of overweight and obesity [4, 5].

Recent reviews indicate that parental involvement and parental monitoring of child health behaviors are important factors to consider in preventing and treating childhood obesity [6]. Some investigators have argued that the home environment is an important setting for shaping children's eating and physical activity (PA) behaviors and that parents are powerful change agents [7]. As such, primary and secondary prevention efforts are needed to focus on the treatment of obesity by altering the perceptions, attitudes, and behaviors of parents who influence their children's diet and energy expenditure [8, 9]. The purpose of the present study was to evaluate parent factors that may contribute to adolescent overweight. Parent factors including parental nurturance, parental limit setting of sedentary behavior, and parental perceived risk for development of T2DM were evaluated as predictors of adolescent zBMI. Understanding parenting-related factors of childhood obesity will help in directing future interventions for preventing overweight.

Family systems theory (FST) provides a framework for understanding how families and parents may influence youth health behaviors. According to FST, functional families are more able to manage daily life in the context of warm and supportive family interactions [10]. Parenting styles that are authoritative having moderate levels of control and high levels of support result in more positive family function including better communication, problem solving, and conflict resolution and have been associated with a range of positive adolescent outcomes [11, 12]. Locke and Prinz [13] consider the dimensions and measurement of parental nurturance and discipline as key parent-related factors in youth development. Parental nurturance has been shown to be associated with a variety of health-related behaviors, including higher levels of fruit and vegetable intake [14], positive body satisfaction, and self-esteem [15], and with more frequently eating breakfast [14]. Taken together the above studies suggest that parental nurturance may serve as an important dimension of the familial context and has an important role in family and child health practices.

Screen time is also considered a substantial contributor to overweight in youth. The American Academy of Pediatrics recommends that screen time for youth be limited to 1-2 hours per day [16]. Nonetheless, youth aged 12–17 years watch over 24 hours of television per week [17]. In a study by Andersen et al. [18] youth who watched four or more hours of television per day were found to have greater body fat and higher BMI than those who watched less than two hours per day. In addition, ethnic minorities exhibited significantly higher levels of television viewing and lower rates of vigorous physical activity (PA). Parental limit setting of screen time may be one important intervention avenue. The current study seeks to examine limit setting in the context of other parent-related variables—such as nurturance—thought to be important in the context of pediatric obesity.

A hallmark clinical trial—the Diabetes Prevention Program (DPP)—found that high-risk individuals (such as those who are overweight) can implement lifestyle changes to avoid the development of T2DM [19]. Unfortunately, research has shown that parents often do not perceive their child as overweight (the most significant risk factor for T2DM) or at risk for health problems such as T2DM, despite the contrary [20], and thus may fail to implement those critical changes. Recently, however, the concept of risk perceptions has begun to be explored more thoroughly and recognized as influential in both preventing and treating overweight in youth [21]. The literature supports the idea that parents often underestimate their child as obese; low parental recognition of overweight status has been replicated across studies [8, 22, 23]. This suggests that those at greatest risk for obesity are also at greatest risk for failing to seek treatment or engaging in active health promotion effort for their youth [24]. In addition to parental nurturance and limit setting, in order to initiate and maintain family behavior change parents must likely also perceive their child to be at risk for negative health consequences. No known study to date has examined the association of parental risk perceptions with other parent related variables (limit setting, nurturance) thought to be related to pediatric obesity.

The goal of this study was to expand on past research by evaluating whether parental nurturance, limit setting, and perceptions of adolescent risk are associated with adolescent zBMI. Specifically, this study examined the associations of parental risk perceptions for their adolescent's development of T2DM, parental limit setting of sedentary behavior, and parental nurturance with adolescent zBMI in a primarily African American population. It was hypothesized that higher levels of parental risk perceptions, limit setting, and nurturance would be associated with lower adolescent zBMI.

## 2. Methods

### 2.1. Participants

This research project was undertaken as part of two studies examining family health (see [25–27] for related studies) with the goal of obtaining a sample with variation in adolescent weight status and sex, as well as ethnic minorities given the increased risk observed among underserved populations for obesity. Families were recruited from two small southeastern communities in South Carolina through community partners, radio, and newspaper advertisements. Families were eligible to participate if they had (1) an adolescent aged 11 to 15 years, (2) at least one parent living in the same household as the adolescent willing to participate, and (3) no physical or dietary restrictions. A total of approximately 350 families were contacted, resulting in seventy parent-adolescent dyads (see Table 1).

### 2.2. Procedures

The Institutional Review Board at the University of South Carolina approved the study prior to enrolling participants. Parents signed an informed consent and adolescents signed an assent form to participate. Demographic information was obtained from parents, and both the parents and adolescents completed psychosocial surveys and anthropometric measures of height and weight.

### 2.3. Measures

A Shorr Height measuring board was used to obtain height measurements, and weight was measured with a SECA 880 digital scale. Two measures of height and weight were taken by certified study staff members for both adolescents and their parents. The average was then computed and utilized in BMI calculations. Indices of the anthropometric status of adolescents (z-score for body mass index-for-age, BMI values, and BMI-for-age percentiles) were calculated based on the 2000 CDC growth charts and a Statistical Analysis System (SAS) program made available by the CDC [28]. Parent BMI was also calculated based on the standard formula of weight (kg)/height (m)^2^.

Parental risk perceptions of T2DM were assessed through a modified version of the Risk Perception Survey for Developing Diabetes (mRPS-DD; [29]). For the current study, the RPS-DD was modified to reflect parent responses to the items based on the risk perceptions for their child. Parents were asked to respond to these items reflecting on their attitudes and behaviors towards the child participating in the current study. A 4-point Likert response format, ranging from strongly disagree to strongly agree, was used to measure parent's level of agreement with each statement. The Worry subscale of the mRPS-DD was used for the purpose of the present study. During the survey development phase of the original RPS-DD, items were reviewed by a panel of clinical experts, including health psychologists, for face and content validity. Internal consistency reliability, as reflected by coefficient alpha, has ranged from 0.65 to 0.80 [29, 30]. The instrument has been used in previous studies [29–32], including the DPP trial [19] to examine risk perceptions of T2DM.

The Limiting-Activity subscale of the previously validated Parenting Strategies for Eating and Activity Scale (PEAS) [33–35] was used to assess parents' use of appropriate boundaries for sedentary behavior. The reliability of the Limiting subscale has been shown to be adequate, with an internal consistency of 0.81–0.87 [36, 37]. For the current study, the coefficient alpha value for this subscale was 0.76. A 4-point Likert response format was used to assess parent's responses to the Limiting-Activity subscale of the PEAS. Parents were instructed to indicate how often they engage in the particular parenting practice specified in each item. Each item response ranged from 1: “Strongly Disagree” to 4: “Strongly Agree.”

The Parenting Dimensions Inventory-Short Version (PDI-S) [38], a 27-item self-report instrument, was administered to parents. The PDI-S measures several dimensions of parenting, including parental support, parental control, and parental structure. For the purposes of this study, only the parental nurturance subscale was utilized as a primary construct of interest. The nurturance subscale of the PDI-S has a total of 6 items which measure emotional nurturance, focusing on emotional expressions of warmth and support, such as verbal statements of love, communication of acceptance, and physical affection and warmth [13]. The reliability of the nurturance subscale has been shown to be adequate with an internal consistency of 0.80 [36] in the original sample. The reliability of this subscale for the current sample was 0.76. Moreover, the subscale has shown high stability over a four-year period (*r* = 0.46, *P* < 0.0001; [38]). A 6-point Likert format was used and parents were asked to choose the response that mostly closely applied to them and their child, with responses choices ranging from 1: “Not at all like me” to 6: “Exactly like me.”

### 2.4. Data Analyses

Data were reduced and analyzed using SPSS Statistics software, version 17.0, and SAS software, version 9.0. The data were analyzed for outliers, normality, missing values, and linearity. Sex (male/female) was recoded as a dummy variable. Variables, excluding variables that were dummy coded or already standardized (i.e., zBMI), were centered to enhance beta weight interpretability. An inverse square root transformation was conducted on the outcome variable due to concerns regarding normality of the distribution [37, 39]. This transformation resulted in an improvement in the skewness (−0.306) but increased the flatness of the distribution (kurtosis = −1.050). The Kolmogrov-Smirnoff test of normality was nonsignificant when considering this transformed distribution, *P* > 0.05, indicating improvement in the normality of the distribution.

Pearson product moment correlations were used to analyze the associations among variables. A multiple linear regression model was conducted to determine if adolescent zBMI could be predicted from parental factors (including risk perceptions, limit setting, and parental nurturance) while controlling for adolescent sex, age, and parent weight status (variables which have been highlighted as risk factors for pediatric obesity). An additional, post-hoc analysis was conducted to explore whether parental limit setting moderates the relationship between adolescent zBMI and parental nurturance. A simple moderated regression was conducted to ascertain whether or not this relationship was significant; simple slopes analysis was not conducted due to the lack of significance. Significance level for this study was defined as *P* ≤ 0.05.

## 3. Results

### 3.1. Demographic and Descriptive Variables

Table 1 provides a summary of the study sample demographics, including pertinent adolescent, parent, and family characteristics. Additionally, the means, standard deviations, and range of scores for parent variables were calculated. The mean parental nurturance score (*M* = 4.99, SD = 0.85) suggested that parents self-reported exhibiting moderate levels of warmth and support. The average limit-setting score (*M* = 3.31, SD = 0.75) revealed that parents reported moderate-to-high levels of engagement in parenting practices related to limit setting. Finally, the mean parental risk perceptions score (*M* = 2.87, SD = 0.77) was slightly lower, indicating that on average parents reported not often worrying about the risks of T2DM for their youth.

### 3.2. Correlational Analyses

Pearson product moment correlations (*r*) among adolescent zBMI, adolescent age, parent BMI, parent risk perceptions, parent limit setting, and parental nurturance revealed a significant positive correlation between adolescent zBMI and parental risk perceptions (*r* = 0.327, *P* < 0.01), indicating that as adolescent zBMI increased, parental perceptions of diabetes risk also increased. In addition, a significant positive correlation between adolescent zBMI and parent BMI was found (*r* = 0.301, *P* ≤ 0.05), showing that the more overweight the parent, the higher the adolescent zBMI. No other significant correlations were reflected.

### 3.3. Multiple Regression Analyses

Multiple linear regression analysis (see Table 2) was conducted to evaluate whether parent factors significantly predicted adolescent zBMI while controlling for adolescent age, gender, and parent BMI. No multivariate outliers were detected using Cook's Distance. Tolerance, as a measure of collinearity, was acceptable with values ranging from 0.81 to 0.99, and the Variance Inflation Factor (VIF) did not exceed 1.2, well below the standard criteria for violation value of 10. The linear combination of predictor variables was significantly related to adolescent zBMI, (6, 63) = 4.38, *P* = 0.001. Approximately 23% of the variance (adjusted *R* ^2^ = 0.23) in adolescent zBMI in the sample was accounted for by the overall model. In addition, a significant beta coefficient was demonstrated for parental nurturance (*β* = 0.30, *P* < 0.01) reflecting a positive relationship with adolescent zBMI, such that higher levels of reported nurturance were associated with higher zBMI values. Parental limit setting was also a significant predictor (*β* = −0.24, *P* < 0.05), with lower levels of limit setting associated with higher zBMI values. Lastly, parental risk perceptions was positively related to adolescent zBMI, such that higher levels of parental perceptions of risk for T2DM associated with higher zBMI values (*β* = −0.25, *P* < 0.05).

**Figure 1:**
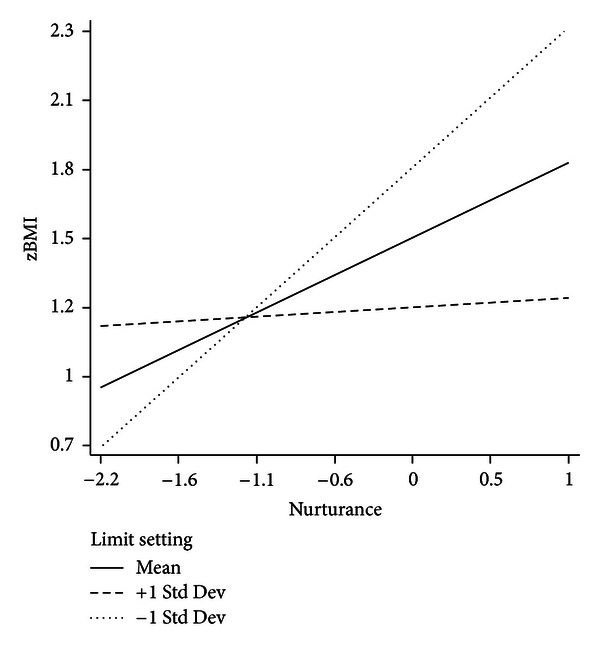
Model of the moderational role of parental limit setting in the relationship between parental nurturance and adolescent zBMI.

As the relationship between parental nurturance and adolescent zBMI was in the direction opposite of what was hypothesized, a post-hoc analysis was conducted to explore the potential relationship between parent nurturance and parent limit setting. Specifically, the idea that the influence of family nurturance on adolescent zBMI varies as a function of parental limit setting was explored. Parental nurturance, limit-setting, and the interaction term were entered into the regression equation and the interaction term approached significance (*P* = 0.079). At higher levels of parental limit-setting and higher levels of nurturance, adolescent zBMI values were lower; however, at lower levels of parental limit setting but higher levels of nurturance, adolescent zBMI values were higher (Figure 1).

## 4. Discussion

This study investigated parent factors associated with adolescent overweight, conceptualizing the family as central to the etiology and maintenance of pediatric obesity [40]. Parental risk perceptions, limit setting, and nurturance were all significantly related to adolescent zBMI in the present study when controlling for adolescent age, gender, and parent weight status. The results of the current study provide some insight into important parental variables that are related to adolescent overweight and may be important to incorporate for future intervention treatment programs for overweight adolescents.

In the present study parental limit setting of sedentary behavior was a significant predictor of adolescent zBMI. Setting limits on sedentary behavior has been cited as an important area of opportunity for intervention efforts [41]. Limit setting may operate both directly and indirectly as it may increase PA and has also been shown to improve self-regulation [42], which plays an important role in preventing overweight [43]. Israel et al. [44] found that interventions targeted at enhancing self-regulation were directly related to decreased body fatness. As such, parental limit setting will be an important factor for future interventions with both direct and indirect benefits to adolescent weight-status.

Interestingly, parental nurturance was a significant predictor of adolescent zBMI in the current study but in the direction contrary to proposed hypotheses. Though much of the literature has focused on the benefits of parents who exhibit warmth and nurturance, [18, 45], the observed effect in the present study was not in the hypothesized direction as higher levels of nurturance were associated with higher adolescent zBMI values. A post-hoc analysis was conducted to determine whether the influence of parental nurturance on adolescent zBMI varied as a function of parental limit setting. This interaction effect did not reach statistical significance; however, this trend is of interest given the small sample size of the current study. This trend suggests that as the level of parental limit setting increased, the positive relationship between nurturance and zBMI weakened. More specifically, high levels of nurturance and high levels of parental limit-setting were associated with lower zBMI values. This may be interpreted in light of a line of research from previous studies that have examined indulgent parenting and feeding styles characterized by high nurturance and low structure, which have been linked to increased body mass in children [46–48]. This suggests the possibility of an optimal combination of parental nurturance and parental limit-setting in which parents are sensitive and caring but also provide their children with guidelines that provide structure, particularly around sedentary behavior. Further research should be conducted in this area to establish whether parental limit-setting moderates the effect of parental nurturance on adolescent zBMI and to, more generally, continue to clarify the links between parenting style and children's health behaviors.

There are several strengths of this study including a primarily ethnic minority sample of participants and an examination of a set of modifiable parent-related variables. Few studies focusing on adolescent and overweight have considered ethnic minorities, and fewer ones still have incorporated an array of key familial variables [49, 50] however, future work should seek to determine whether the current results hold across other populations. The current study took a family systems perspective, acknowledging the multifactorial nature of pediatric obesity. Several limitations of the current study should also be noted. A limitation of the current study was the small sample size and cross-sectional nature of the study design. Future studies should incorporate larger sample sizes and a longitudinal approach. For instance, it may be informative for future longitudinal studies to examine the development of parental risk perceptions across time to better understand the critical points at which prevention efforts may be most effective. In addition, the study targeted overrecruiting an underserved ethnic minority population, and thus, the sampling method was not completely at random.

In summary, this study supports the notion that parental perceptions of risk, limit setting, and nurturance may be important in understanding pediatric obesity and should be considered for future interventions. In forecasting the future burden of current adolescent overweight in the United States, Lightwood et al. [51] predict that overweight in our society will have dramatic implications both in humanistic terms, considering the impact on quality of life and premature death, and in fiscal terms, considering the heavy economic burdens. The current study presents a set of key parent-related variables that had yet to be examined in combination, particularly in a predominantly African American sample. The examination of parental risk perceptions for T2DM risk is especially noteworthy, as there is a gap in the research in this area. As research has continued to show the importance of taking a family systems approach to the obesity epidemic, studies should persist in investigating the complex condition of obesity, taking a family systems approach, and considering combinations of variables to inform primary T2DM and secondary obesity prevention practices. Approaches that incorporate a family systems approach will serve to lead the field in providing a more comprehensive analysis of the obesity epidemic, thereby informing clinical practice.

## Figures and Tables

**Table 1:** Sample demographic characteristics (*N* = 70 parent-adolescent dyads).

Variable	Statistic
Adolescent mean age (SD)	12.6 (1.34)
Adolescent gender (%): female	58.6
Adolescent weight status (%)	
Normal	30.0
Overweight	18.6
Obese	51.4
Ethnicity (%): African American	90
Parental marital status (%): married	45.7
Parent mean BMI (SD)	35.2 (7.61)
Parent weight status (%)	
Normal weight	7.1
Overweight	18.6
Obese	74.3
Family history of diabetes (%): yes	37.1
Family history of hypertension (%): yes	77.1
Highest level of education completed (%) by parent	
Grades 9–11 (some high school)	7.1
High school graduate	21.4
College 1 year to 3 years	38.6
College graduate	20
Graduate training or professional degree	12.9
Gross household yearly income (%)	
Less than $10,000	14.3
$10,000–24,000	28.6
$25,000–39,000	17.1
$40,000–54,000	17.1
$55,000–69,000	4.3
$70,000–84,000	7.1
$85,000 or more	7.1
Other	4.3

**Table 2:** Multiple regression analysis of parental variables predicting adolescent zBMI.

Variable	*B*	SE	*β*	*P*	*F*	*R* ^2^
					4.378	0.227
Adolescent sex	0.250	0.198	0.138	0.211		
Adolescent age	−0.010	0.006	−0.176	0.117		
Parent weight status	0.364	0.161	0.245	0.027*		
Parental nurturance	0.363	0.133	0.301	0.008**		
Parental limit setting	−0.255	0.120	−0.239	0.037*		
Parental risk perceptions	−0.263	0.130	−0.225	0.047*		

*P ≤ 0.05, **P ≤ 0.01.
